# Zoonotic disease preparedness in sub-Saharan African countries

**DOI:** 10.1186/s42522-021-00037-8

**Published:** 2021-03-22

**Authors:** Linzy Elton, Najmul Haider, Richard Kock, Margaret J. Thomason, John Tembo, Liã Bárbara Arruda, Francine Ntoumi, Alimuddin Zumla, Timothy D. McHugh, And the PANDORA-ID-NET consortium

**Affiliations:** 1 Centre for Clinical Microbiology, Division of Infection & Immunity, University College London, London, UK; 2 Royal Veterinary College, London, UK; 3 University of Lusaka, Lusaka, Zambia; 4 Institute for Tropical Medicine, University of Tübingen, Tübingen, Germany; 5 Congolese Foundation for Medical Research, Brazzaville, Republic of Congo; 6 National Institute for Health Research Biomedical Research Centre, University College London Hospitals NHS Foundation Trust, London, UK

**Keywords:** Zoonotic disease, Joint external evaluation, One health, Sub-Saharan Africa

## Abstract

**Background:**

The emergence of high consequence pathogens such as Ebola and SARS-CoV-2, along with the continued burden of neglected diseases such as rabies, has highlighted the need for preparedness for emerging and endemic infectious diseases of zoonotic origin in sub-Saharan Africa (SSA) using a One Health approach. To identify trends in SSA preparedness, the World Health Organization (WHO) Joint External Evaluation (JEE) reports were analysed. JEEs are voluntary, collaborative processes to assess country’s capacities to prevent, detect and rapidly respond to public health risks. This report aimed to analyse the JEE zoonotic disease preparedness data as a whole and identify strengths and weaknesses.

**Methods:**

JEE zoonotic disease preparedness scores for 44 SSA countries who had completed JEEs were analysed. An overall zoonotic disease preparedness score was calculated as an average of the sum of all the SSA country zoonotic disease preparedness scores and compared to the overall mean JEE score. Zoonotic disease preparedness indicators were analysed and data were collated into regions to identify key areas of strength.

**Results:**

The mean ‘Zoonotic disease’ preparedness score (2.35, range 1.00–4.00) was 7% higher compared to the mean overall JEE preparedness score (2.19, range 1.55–3.30), putting ‘Zoonotic Diseases’ 5th out of 19 JEE sub-areas for preparedness. The average scores for each ‘Zoonotic Disease’ category were 2.45 for ‘Surveillance Systems’, 2.76 for ‘Veterinary Workforce’ and 1.84 for ‘Response Mechanisms’. The Southern African region scored highest across the ‘Zoonotic disease’ categories (2.87).

A multisectoral priority zoonotic pathogens list is in place for 43% of SSA countries and 70% reported undertaking national surveillance on 1–5 zoonotic diseases. 70% of SSA countries reported having public health training courses in place for veterinarians and 30% had veterinarians in all districts (reported as sufficient staffing). A multisectoral action plan for zoonotic outbreaks was in place for 14% countries and 32% reported having an established inter-agency response team for zoonotic outbreaks. The zoonotic diseases that appeared most in reported country priority lists were rabies and Highly Pathogenic Avian Influenza (HPAI) (both 89%), anthrax (83%), and brucellosis (78%).

**Conclusions:**

With ‘Zoonotic Diseases’ ranking 5th in the JEE sub-areas and a mean SSA score 7% greater than the overall mean JEE score, zoonotic disease preparedness appears to have the attention of most SSA countries. However, the considerable range suggests that some countries have more measures in place than others, which may perhaps reflect the geography and types of pathogens that commonly occur. The category ‘Response Mechanisms’ had the lowest mean score across SSA, suggesting that implementing a multisectoral action plan and response team could provide the greatest gains.

## Study highlights

- ‘Veterinary Workforce’ was the strongest ‘Zoonotic Disease’ category across SSA
- ‘Response Mechanisms’ was the weakest category, and improving communication between clinical, veterinary and environmental sectors could improve zoonotic disease responses
- Southern Africa had the highest mean score for all ‘Zoonotic Disease’ categories, suggesting that other countries could adapt their strengths to their own situations
- The 5 most-cited zoonoses on SSA priority pathogen lists are rabies, Highly pathogenic avian influenza (HPAI), anthrax, brucellosis and bovine tuberculosis, all of which are ‘neglected’ diseases

## Introduction

Many human infectious diseases have originated from animals. It is thought that 60% of currently known human infectious diseases [1] and as much as 75% of emerging infectious diseases are of zoonotic origin, or the result of a spill over event which then established itself in humans [1, 2]. Whilst many high income countries (HICs) have successfully reduced or eradicated zoonoses, often utilizing expensive interventions, the heaviest burden of zoonotic diseases now often falls on low and middle-income countries (LMICs), who historically have the poorest healthcare infrastructure and rely most heavily on livestock economically [3]. This is especially important in sub-Saharan Africa (SSA), where reliance on livestock and bush meat can be high [4]. The fact that, in many of the regions affected by zoonotic diseases, livestock play a vital role both as a cash reserve and source of income in poor communities makes it not only medically, but also economically, important to ensure animals are kept healthy [4]. The environmental and social change our expanding population is causing is affecting the way infectious diseases spread across the globe. The increase in air travel to and from high-burden areas such as South America, Africa and Asia is likely to increase the introduction of vector-borne pathogens to new regions, whilst the abundance of human-commensal species, such as rats, is increasing alongside urbanisation and deforestation, raising the opportunity for human transmission of zoonoses [5].

As, by definition, zoonotic diseases are infectious diseases that are naturally transmitted from vertebrate animals to humans and vice versa [6], the need for a One Health approach cannot be overstated. Limiting or eliminating the transfer of pathogens between animals, the environment and humans, and therefore reducing the risk of them becoming an emerging infectious disease is of vital importance. The value of zoonotic pathogen surveillance is beginning to gain traction in Africa, although it is often difficult to identify whether data are collected, never mind what trends may exist [7–9]. The World Health Organization (WHO) notes that the steps to achieve a One Health approach should include: promoting the concept of a multisectoral approach and developing integrated control packages, raising the profile of neglected zoonotic diseases, collecting surveillance data to identify those at risk, and investing in the development of new tools, particularly diagnostics, to effectively control zoonoses [4, 10].

Zoonotic disease spill over events (when zoonotic pathogens are transmitted to humans) occur frequently in Africa, including direct zoonotic events, from animals to humans, such as Lassa fever across West Africa (2016–2018) [11] and Monkeypox in West and Central Africa [12], as well as secondary, human-to-human epidemiological cycles such as Ebola in West Africa (2013–2016) and the Democratic Republic of Congo (2019) [13, 14]. There are also more localised, but common, outbreaks of diseases such as salmonellosis and Yellow Fever [15]. Some, such as Rift Valley Fever, can occur locally, such as in South Africa [16] or be more widespread, such as across Eastern Africa, as well as the Middle East [17].

As a first step in preventing emerging infectious disease outbreaks of animal origin in humans, it is important for SSA countries to know what level of support systems are already in place. The scientific literature mainly focuses on the research being done, rather than the infrastructure that supports it, but reports from global organisations such as the Africa Centre for disease Control (Africa CDC), US Centers for Disease Control (US CDC), WHO, World Organisation for Animal Health (OIE) and the Food and Agriculture Organisation (FAO) suggest that much more could be done to combat zoonoses [4, 18–20]. The WHO reports that, due to multiple factors such as poor funding, a lack of veterinary and clinical cooperation and disease misdiagnosis, the real burden of zoonoses is often missed [4]. This creates a lack of reliable evidence for governments and policy makers to utilise when implementing legislation at both local and national levels [21]. This WHO report also suggests that control measures are often undertaken in isolation, which is therefore likely to make them less effective than when part of a suite of control measures [4].

Strengthening country-specific preparedness for potential public health risks, including zoonotic disease, has increasingly become the focus for governments and health organisations. The 58th World Health Assembly in 2005 resulted in the formation of the International Health Regulations, which developed the Joint External Evaluations (JEEs): voluntary, collaborative procedures that assess a country’s capacity to ‘prevent, detect and rapidly respond to public health risks’ [22]. The zoonotic disease preparedness for countries is measured by the presence of a ‘functional multisectoral, multidisciplinary mechanisms, policies, systems and practices to minimize the transmission of zoonotic diseases from animals to human populations’ [23]. In March 2020, 86% (44/50) of the SSA countries (according to WHO definition), had completed a JEE [23, 24].

JEE reports cover prevention, detection, response and IHR related hazards and points of entry areas, which are classified into 19 sub-areas. ‘Zoonotic Disease’ is one of these sub-areas and is divided into three categories. Technical questions and indicators scores are applied to evaluate the preparedness of a country to a specific zoonotic disease. The indicator scores range from 1 to 5, indicating if a country has respectively: no, limited, developed, demonstrated or sustainable capacity to address a particular sub-area.

Whilst there are many areas of zoonotic disease to focus on, it would be beneficial to step back and look at the wider picture. A comparison of the zoonotic disease facilities and infrastructure across SSA has yet to be made, and using JEE’s provides a robust, standardised set of indicators to detect stronger and weaker areas across SSA. This provides an outline of how SSA is tackling zoonotic diseases, and how well zoonotic disease preparedness is implemented compared to other public health concerns, such as antimicrobial resistance. When collated into African regions (West, Central, East and Southern), these strengths can be further focussed and adapted by countries who may be finding it more challenging. The data acquired from this study can inform zoonotic disease policy in the future. This study aimed to analyse the JEE data to understand zoonotic disease preparedness across SSA countries.

## Methods

The completed JEE reports from 44 SSA countries (as described by the WHO regions), were accessed between 6th November 2019 and 22nd March 2020 and analysed [25] and the sub-areas scores were compared [24]. The analysis of these data is described in Elton et al. [24]. The ‘Zoonotic disease’ sub-area was compared to the other sub-areas (such as immunization and antimicrobial resistance preparedness) and a ranking table was compiled [24].

The percentage of countries in each score category was determined, as well as the percentage of the countries that reported having zoonotic disease prevention and control structures in position, which was identified from the accompanying technical question written reports, as described in Table 1. In the first edition of the JEE tool [26], there were three categories within ‘Zoonotic Disease’, whereas by 2018, the second edition [22] had removed the category ‘Veterinary or animal health workforce’. The majority (86%) of countries had completed JEEs before this change, thus have all three categories (Central African Republic, Republic of Congo, Malawi, Gabon, Guinea Bissau, São Tomé and Príncipe and Comoros were completed later and have two categories). These scores were weighted into regions (15 West, 7 Central, 17 East and 5 Southern African countries, as defined by the United Nations) to pinpoint any patterns of zoonotic disease preparedness strengths [27]. The guidelines for how scores are ascribed are outlined in the JEE Tool [22]. Data was analysed as described in [24].

**Table 1:** Zoonotic disease preparedness categories and the indicators assessed. Indicators are taken from the scoring table (see the JEE tool, second edition [25]) or a technical question answer from the written report

Category	Indicator	Source
Surveillance systems in place for priority zoonotic diseases/pathogens (in this paper referred to as ‘Surveillance Systems’) (this section is included in the JEE tool first and second editions)	Is there a national surveillance system in place for up to 5 priority zoonotic diseases?	Scoring table within the JEE tools document
	Has a multisectoral agreement on 5 priority zoonotic pathogens been made?	Technical questions
	Is there a national surveillance system in place for more than 5 zoonotic diseases?	Technical questions
	Do clinical and veterinary laboratories communicate data/results with each other?	Technical questions
Veterinary or animal health workforce (in this paper referred to as ‘Veterinary Workforce’) (this section is in the JEE tool first edition only)	Is there an animal health workforce capacity within the national public health system and at all sub-national levels?	Scoring table within the JEE tools document
	Are there training opportunities for veterinary workers in zoonotic disease and transmission?	Technical questions
Mechanisms for responding to infectious and potential zoonotic diseases are established and functional (in this paper referred to as ‘Response Mechanisms’) (this section is included in the JEE tool first and second editions)	Is an established multisectoral operational action plan for coordinated response to outbreaks of zoonotic diseases in place?	Scoring table within the JEE tools document
	Do you have an established inter-agency zoonotic disease response team?	Technical questions
	Do you have the capacity to respond to 80% of zoonotic events in a timely manner?	Technical questions

### Findings

When the SSA mean score for the sub-area ‘Zoonotic Disease’ was compared to the other sub-areas, it scored 2.35, placing it 5th out of 19 (range 1.33–3.38; *p* < 0.0001). The sub-area ‘Immunization’ ranked highest, with an average SSA score of 3.38, whilst ‘Medical countermeasures and personnel deployment’ scored lowest, with a SSA average of 1.33.

**Fig. 1:**
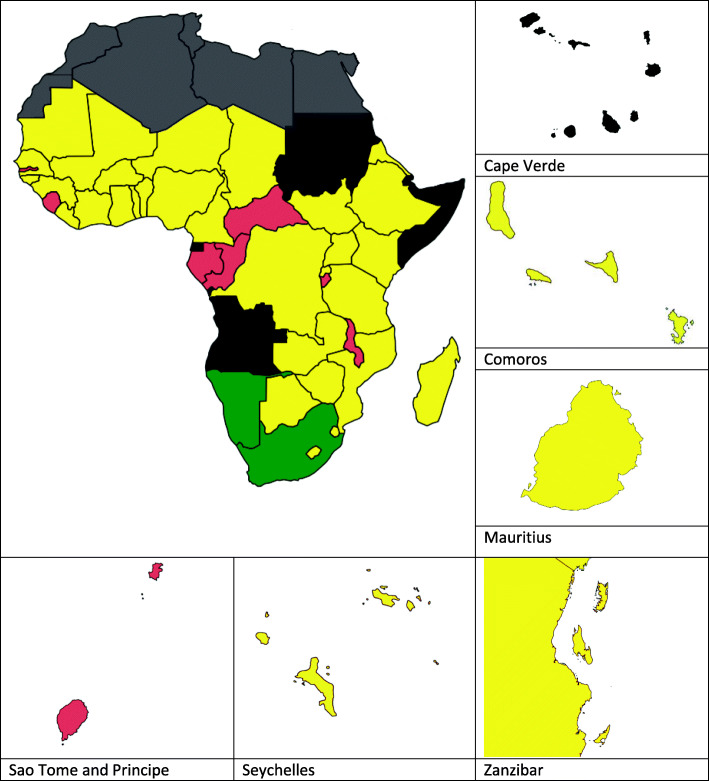
Map showing SSA country mean zoonotic disease preparedness scores. Red = a score of 1 (‘no capacity’). Yellow = 2 or 3 (‘limited or developed capacity’) and green = a score of 4 or 5 (‘demonstrated capacity’ or ‘sustainable capacity’). Countries that have not completed a JEE report are shown in black and North African countries not included in this review are coloured grey. For the countries included in this study, please see [27]

The mean score for all SSA countries for ‘Zoonotic Disease’ was 7% higher than the mean overall JEE preparedness score for SSA. Figure 1 shows the mean ‘Zoonotic Disease’ score for each country by colour category, as described in the JEE tool document.

When the mean for SSA countries was weighted by ‘Zoonotic Disease’ category, there was a significant difference between the mean SSA category scores (*p* = 0.0006) (Table 2). ‘Veterinary Workforce’ had the highest score of 2.76 (range 1.00–4.00). The category ‘Response Mechanisms’ had the lowest averaged score of 1.84 (range 1.00–4.00).

**Table 2:** Percentage of countries according to score and category. The *p* value indicates significant difference between the categories

Score (capacity)	Surveillance Systems	Veterinary Workforce	Response Mechanisms
**1 (no)**	8 (18%)	3 (7%)	17 (40%)
**2 (limited)**	13 (30%)	11 (27%)	18 (43%)
**3 (developed)**	19 (43%)	16 (39%)	8 (19%)
**4 (demonstrated)**	3 (7%)	8 (20%)	1 (2%)
**5 (sustainable)**	1 (2%)	0 (0%)	0 (0%)
**Mean score**	2.45	2.76	1.84
***p*** **value**	*p* = 0.0006		

Each zoonotic disease preparedness indicator had a score, but the depth of written responses to the questions varied. In the ‘Surveillance Systems’ category 70% SSA countries reported undertaking surveillance for between 1 and 5 zoonotic diseases and 27% reported undertaking surveillance for more than 5 zoonotic diseases. A list of priority pathogens, agreed across health and veterinary sectors, is in place for 43% SSA countries and 23% SSA countries indicated that their clinical and veterinary laboratories/departments communicated with each other.

In the ‘Veterinary Workforce’ category, 30% reported having veterinarians in all districts, thus having sufficient country-wide veterinary staffing and 70% of countries reported having training courses for their veterinarians and animal health workers in public health.

In the category ‘Response Mechanisms’, 14% of SSA countries reported having a multisectoral National Action Plan in place for zoonotic diseases, whilst 32% of countries reported having an established inter-agency response team for zoonotic diseases. Fewer (9%) reported having the capacity to respond to 80% of zoonotic events on time (although this indicator had the fewest responses, with 55% of countries not making reference to it in the written report). The SSA country responses to the zoonotic disease preparedness indicators are described in Table 3.

**Table 3:** The percentage of countries stating that they had an indicator established

Category	Region (Number of countries)	All SSA countries	West	Central	East	Southern
		(44)	(15)	(7)	(17)	(5)
Surveillance	Surveillance system for ≤5 diseases	34 (77%)	11 (73%)	7 (100%)	13 (76%)	3 (60%)
	List of 5 priority zoonotic pathogens	19 (43%)	6 (40%)	2 (29%)	8 (47%)	3 (60%)
	Surveillance system for > 5 diseases	13 (30%)	4 (27%)	2 (29%)	4 (24%)	3 (60%)
	Clinical and veterinary communication	11 (25%)	4 (27%)	1 (14%)	5 (29%)	1 (20%)
Veterinary Workforce	National veterinary workforce capacity	13 (30%)	3 (20%)	1 (14%)	6 (35%)	3 (60%)
	Veterinary worker training	31 (70%)	11 (73%)	3 (43%)	12 (71%)	5 (100%)
Response Mechanisms	Established multisectoral action plan	6 (14%)	0 (0%)	2 (29%)	3 (18%)	1 (20%)
	Inter-agency zoonotic response team	15 (34%)	6 (40%)	2 (29%)	6 (35%)	1 (20%)
	Capacity to respond to 80% of events	4 (9%)	0 (0%)	1 (14%)	3 (18%)	0 (0%)

When category scores were weighted by region, it showed that Southern Africa had the highest mean score across all ‘Zoonotic Disease’ categories (range: 1.71–2.87). Table 4 shows the regional mean scores for each of the ‘Zoonotic Disease’ categories, as well as mean ‘Zoonotic Disease’ category and overall JEE mean scores. The regions showed significant differences in all categories except ‘Response Mechanisms’.

**Table 4:** SSA mean zoonotic preparedness category scores by region. Southern Africa had the highest mean ‘Zoonotic Disease’ score. All categories except ‘Response Mechanisms’ showed significant differences in scores between regions

Total countries (in SSA)		Surveillance	Veterinary Workforce	Response Mechanisms	‘Zoonotic Disease’ category mean	Overall JEE mean
**West**	15	2.00	2.20	1.87	2.02	2.09
**Central**	7	1.57	1.57	2.00	1.71	1.75
**East**	17	2.29	2.59	2.00	2.29	2.38
**Southern**	5	2.40	4.00	2.20	2.87	2.40
***p*** **value**		*p* = 0.0017	*p* = 0.0036	*P* = 0.3947	*p* = 0.0006	*p* = 0.0074

When assessing the preparedness indicators, Southern Africa had the highest percentage of countries with an approved list of priority pathogens (60%), surveillance in place for > 5 zoonotic diseases (60%), sufficient veterinarians in all districts (60%) and public health training for veterinary and animal health practitioners (100%). Central Africa had the highest percentage of countries reporting surveillance for between 1 and 5 zoonotic diseases (100%) and having multisectoral National Action Plans for zoonotic diseases (33%). West Africa had the highest percentage of countries reporting having an established inter-agency zoonotic disease response team (33%) and East Africa had the highest percentage of countries reporting that clinical and veterinary laboratories/services communicated with each other (29%) and having the capacity to respond to 80% of zoonotic events on time (17%).

**Fig. 2:**
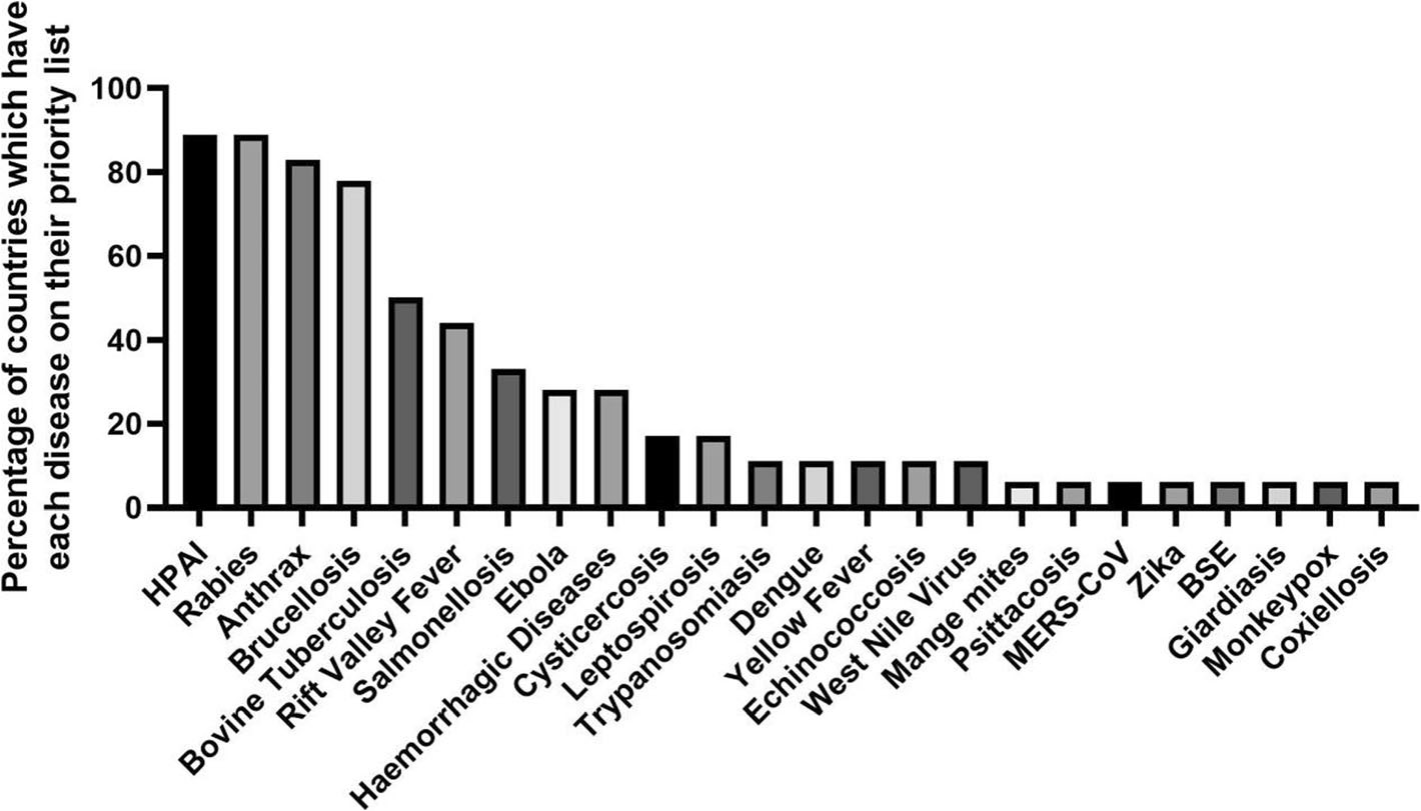
Histogram to show zoonotic diseases by the percentage of countries citing them on their priority pathogen lists. Rabies, Highly Pathogenic Avian Influenza (HPAI), anthrax, brucellosis and bovine tuberculosis were most frequently reported on SSA country priority pathogen lists. MERS-CoV stands for Middle East respiratory syndrome and BSE stands for bovine spongiform encephalopathy. The diseases included in the term ‘Haemorrhagic fevers’ varies between countries

Nineteen countries indicated that they had a multisector-approved list of priority zoonotic pathogens and had listed some or all of those diseases. The zoonotic diseases that appeared most in reported country priority lists were rabies and Highly Pathogenic Avian Influenza (HPAI) (both 89%), anthrax (83%), and brucellosis (78%) (Fig. 2). Whilst some of these are defined as ‘true’ zoonotic diseases, others such as Dengue and SARS are defined as emerging infectious diseases [28]. Twenty three pathogens were reported to be on SSA country priority pathogen lists and a further seven were identified as important zoonotic pathogens (either in countries without an approved priority list, or as non-priority but still worthy of surveillance).

## Discussion

JEEs are an effective way to highlight a country’s ability to address global health issues, including zoonotic diseases and outbreaks. That the mean ‘Zoonotic Disease’ score for SSA countries is higher than the overall JEE score, and that ‘Zoonotic Diseases’ ranks 5th in the JEE sub-areas, suggests that zoonoses and outbreaks are perceived to be of importance by most African countries. However, as there was a larger range in the ‘Zoonotic Disease’ scores compared to the overall JEE score, this suggests that zoonoses are a greater priority in some countries than others. This may reflect the geography, land use, and prevalence and usage of animal species in communities, as well as the donor driven initiatives, which might dictate how often humans come into contact with these zoonotic diseases and therefore how often cases or spill over may occur.

‘Veterinary Workforce’ scored highest of the three ‘Zoonotic Diseases’ categories. This is potentially a very positive aspect of the scores, as having active national disease surveillance and well-trained staff is an excellent basis upon which to build an effective zoonotic disease preparedness system. The category ‘Response Mechanisms’ scored lowest and therefore concentrating on building up these factors, and disseminating to the appropriate veterinary or animal health staff within the national public health system, may quickly improve country scores.

The written reports accompanying the scores gave a deeper insight into the facilities and measures currently in place and yet to be achieved for each SSA country, although this depended on the depth of the response. For the category ‘Surveillance Systems’, whilst three quarters reported that they undertake surveillance for 1–5 zoonotic diseases, under half reported having a list of priority pathogens agreed by all sectors. This suggests that although zoonotic diseases are certainly on the radar of many SSA countries, many do not yet have the regulatory and administrative capacity in place to fully manage the problem, although the process of prioritising has been published by a number of countries, enabling others to follow suit [29, 30]. That only one quarter of countries reported that their clinical and veterinary laboratories communicated with each other suggests that real gains could be made by bringing together the different sectors to create One Health guidelines. The OIE have created a version of the JEEs, evaluating veterinary services, although so far data from these surveys are available for only 36 countries [31]. If the two were to be combined, this would create a much more cohesive response to global health issues.

This is especially true when the fact that, in the ‘Veterinary Workforce’ category, almost three quarters of SSA countries reported having public health training for their veterinary and animal health staff within the national public health workforce, who are capable of following these guidelines. A list of One Health training programmes and resources has been compiled by Rwego et al. (2016) [34]. By increasing the amount of trained veterinary and animal health professionals, and therefore increasing the regional coverage, the lower scoring aspects can be developed and successfully rolled out across a country.

In the ‘Response Mechanisms’ category, one third of countries reported having an established inter-agency response team in place, although only six countries reported having a multisectoral National Action Plan in place. This suggests that whilst many countries are engaging their different departments to tackle zoonotic diseases, more work needs to be done to improve legislation, which should enable a more successful response. The adoption of a resolution with an emphasis on the One Health approach for the successful control of 17 neglected tropical diseases, including zoonoses, at the World Health Assembly in 2013 suggests that there is an international urge to move this forward [32].

When the scores were weighted into regions, countries in the Southern African region had the highest mean ‘Zoonotic Disease’ preparedness score, whilst countries in the Central African region had the lowest. The score for countries in the Southern African region was bolstered by a particularly high ‘Veterinary Workforce’ score compared to the other regions, which suggests that lessons could be learned from this region in terms of the training of veterinary staff in the animal and public health sectors.

That all but one of the top six most reported priority pathogens are well established diseases with a long history of human-animal transmission suggests that SSA has so far struggled to control these zoonoses adequately. Cost effective control measures already exist for several neglected zoonotic diseases such as rabies and brucellosis [4], so the fact that they are on around three quarters of SSA country’s priority pathogen lists suggests that more effective systems may need to be implemented, utilising some of this study’s highlighted points, such as greater communication between veterinary and clinical services.

Other commonly cited zoonoses were salmonella, Ebola, and haemorrhagic diseases (for some countries, this collectively included Ebola). The latter two are classed as emerging infectious diseases, which have jumped from being zoonoses to having human to human transmission. Some countries have to contend not only with well established, ‘neglected’ zoonoses, but also with novel ones, which, in the cases of SARS-CoV-2 and Ebola, can put extreme pressure on already often stretched healthcare and infrastructure systems, especially when a country does not have the capacity to respond effectively. Countries reporting the dual problem of established and emerging zoonoses are the ones who need a robust response system the most and are more likely to require assistance and guidance when it comes to zoonotic disease preparedness.

Control measures, not just for zoonotic diseases but for many other diseases, have historically been undertaken individually or in isolation. Stakeholders must embrace the multifactorial, and multisectoral approach to gain the maximum benefit out of these improvements. Zoonoses and their outbreaks need to be recognised as a One Health problem and, as such, greater cooperation must occur between departments to ensure the problems are approached from all sides. Whilst costs for zoonotic disease interventions may seem costly on top of public health strategies, the long term benefit and overall cost effectiveness is likely to be greater [33].

## Conclusions

As zoonotic outbreaks do not respect political borders, and having identified regions with more developed control strategies, this is an excellent opportunity for countries with more advanced zoonotic disease programmes (such as those in the Southern African region) to help other nations improve, by sharing protocols, strategies and training, thus providing better coverage across the continent, and globally. Groups from the region, e.g. SACIDS (http://www.sacids.org/) should be called upon to tailor their knowledge and share it with the rest of the continent. SSA countries need to fully utilise public health, veterinary and environmental government departments, as well as the advice and fundamental research of both African and global organisations, including One Health consortia, such as the Pan-African network PANDORA-ID-Net (www.pandora-id.net), if they are to build a robust One Health zoonotic disease preparedness response.

## Study highlights

- ‘Veterinary Workforce’ was the strongest category across SSA
- ‘Response Mechanisms’ was the weakest category across SSA, and that improving communication between clinical, veterinary and environmental sectors could improve zoonotic disease responses
- Southern Africa had the highest mean score for all ‘Zoonotic Disease’ categories, suggesting that other countries could adapt their strengths to their own situations
- 30% of SSA countries had veterinarians in all districts
- The 5 most-cited zoonoses on SSA priority pathogen lists are rabies, Highly pathogenic avian influenza (HPAI), anthrax, brucellosis and bovine tuberculosis, all of which are ‘neglected’ diseases

## Data Availability

The datasets analysed during the current study are available in the World Health Organization’s website repository. These datasets were derived from the following public domain resources: https://www.who.int/ihr/procedures/mission-reports/en/

## Abbreviations

SSA: Sub Saharan Africa
JEE: Joint external evaluation
WHO: World Health Organisation
LMIC: Low and Middle Income Country
US CDC: United States Centers for Disease Control
OIE: World Organisation for Animal Health
FAO: Food and Agriculture Organisation
HPAI: Highly Pathogenic Avian Influenza
HIC: High Income Country
IHR: International Health Regulations
